# Horizontal inequity in outpatient care use and untreated morbidity: evidence from nationwide surveys in India between 1995 and 2014

**DOI:** 10.1093/heapol/czx016

**Published:** 2017-04-17

**Authors:** Anamika Pandey, George B Ploubidis, Lynda Clarke, Lalit Dandona

**Affiliations:** 1 Public Health Foundation of India, New Delhi, India; 2 Department of Population Health, London School of Hygiene and Tropical Medicine, London, UK; 3 Centre for Longitudinal Studies, Department of Social Science, UCL–Institute of Education, University College London, UK; 4 Institute for Health Metrics and Evaluation, University of Washington, Seattle, WA, USA

**Keywords:** Concentration index, healthcare use, horizontal inequity, India, older population, outpatient, untreated morbidity

## Abstract

Equity in healthcare has been a long-term guiding principle of health policy in India. We estimate the change in horizontal inequities in healthcare use over two decades comparing the older population (60 years or more) with the younger population (under 60 years). We used data from the nationwide healthcare surveys conducted in India by the National Sample Survey Organization in 1995–96 and 2014 with sample sizes 633 405 and 335 499, respectively. Bivariate and multivariate logit regression analyses were used to study the socioeconomic differentials in self-reported morbidity (SRM), outpatient care and untreated morbidity. Deviations in the degree to which healthcare was distributed according to need were measured by horizontal inequity index (HI). In each consumption quintile the older population had four times higher SRM and outpatient care rate than the younger population in 2014. In 1995–96, the pro-rich inequity in outpatient care was higher for the older (HI: 0.085; 95% CI: 0.066, 0.103) than the younger population (0.039; 0.034, 0.043), but by 2014 this inequity became similar. Untreated morbidity was concentrated among the poor; more so for the older (−0.320; −0.391, −0.249) than the younger (−0.176; −0.211, −0.141) population in 2014. The use of public facilities increased most in the poorest and poor quintiles; the increase was higher for the older than the younger population in the poorest (1.19 times) and poor (1.71 times) quintiles. The use of public facilities was disproportionately higher for the poor in 2014 than in 1995–96 for the older (−0.189; −0.234, −0.145 vs − 0.065; −0.129, −0.001) and the younger (−0.145; −0.175, −0.115 vs − 0.056; −0.086, −0.026) population. The older population has much higher morbidity and is often more disadvantaged in obtaining treatment. Health policy in India should pay special attention to equity in access to healthcare for the older population.

Key Messages

- This study used data from nationwide healthcare surveys to provide evidence on the changing inequity in outpatient care and untreated morbidity for the older population compared with the population under 60 years over the last two decades in India.
- The pro-rich inequity in outpatient care was higher for the older population than the younger population in 1995–96, but by 2014 this inequity became similar.
- The inequity in untreated morbidity was disproportionately concentrated among the poor; more so for the older population than the younger population in 2014.
- Pro-poor inequity in the use of public facilities was higher in 2014, more so for the older population than the younger population.

## Introduction

Equity in healthcare utilization has increasingly being acknowledged by both developed and developing countries as an important intermediate step to achieve the goal of equity in health (Grasdal and Monstad 2011). The decision to seek healthcare is not only guided by need, but is also influenced by the sociodemographic background of individuals that predisposes their use of formal medical care and more so by enabling factors such as capacity to pay (Roy and Chaudhuri 2008; Prinja *et al.* 2012b). The World Health Organization assesses the performance of health systems according to the evidence on the gap in healthcare between the rich and the poor once the need for healthcare is controlled for—horizontal inequity (World Health Organization 2000). The resolutions of World Health Assembly (WHA) from 2005 emphasized that everyone should be able to access healthcare and that access to healthcare should not be subjected to financial hardships (World Health Assembly 2005). The World Health Report 2010 builds upon the resolutions of WHA and aims to assist countries to develop a system of financing that makes healthcare accessible to all (World Health Organization 2010). Given the marked disparity in the access to health services, with the poorest and the most disadvantaged being most affected, India has also recognized equitable access to healthcare for all at an affordable cost as an important goal under the new initiative of universal health coverage (Planning Commission of India 2011). Evidence on horizontal inequity in access to healthcare is thus critical for making healthcare delivery systems more efficient.

This study examined the change in horizontal inequities in outpatient care and untreated morbidity between 1995–96 and 2014 contrasting the older population (aged 60 years or more) with the younger population (under 60 years). The evidence on inequities in healthcare will help in developing a rational policy to provide affordable, accessible and cost effective healthcare to the older population in an increasingly pluralistic healthcare system.

## Data and methods

### Data

We used individual level data from two rounds of the National Sample Survey Organization (NSSO): the survey on healthcare of 1995–96 (52nd round), and the survey on social consumption: health of 2014 (71st round). Both surveys were conducted under the stewardship of the Ministry of Statistics and Programme Implementation, Government of India. Details of the sampling design, survey instruments, and initial findings can be found in the national reports (Ministry of Statistics and Programme Implementation 1998, 2015). Both the surveys collected information on treatment status of each spell of ailment reported in a 15-days reference period for a nationally representative sample of 633 405 and 335 499 individuals of all ages (including deceased members) in NSSO 1995–96 and NSSO 2014 surveys, respectively.

### Measures

Households were divided into quintiles using monthly per capita consumption expenditure (MPCE) adjusted to household size and composition (Deaton 1997). The state-specific adult equivalent mean MPCE was used as a cut-off to categorize households into poor and non-poor. The states in India were classified as less and more developed. Eighteen less developed states include eight empowered action group states (Bihar, Jharkhand, Madhya Pradesh, Chhattisgarh, Uttar Pradesh, Uttaranchal, Odisha and Rajasthan), eight north-eastern states (Assam, Arunachal Pradesh, Manipur, Mizoram, Meghalaya, Nagaland, Sikkim, Tripura), Himachal Pradesh and Jammu and Kashmir (Ministry of Health and Family Welfare 2011a).

We examined horizontal inequity (the extent to which people in equal need for healthcare receive equal treatment, irrespective of their income) in outpatient care, untreated morbidity and use of public facilities for outpatient care comparing the older population aged 60 years or more and the population under 60 years at two time points: 1995–96 and 2014. By doing so, we were able to assess both the within and between-group, as well as over-time changes in inequity. All the reported spells of ailment that were treated on medical advice in the 15-days reference period but not as an inpatient of hospital were classified as outpatient care. If no treatment was ever taken on medical advice for the spell of ailment reported in the 15-days reference period, it was considered as an untreated morbidity in both the surveys to facilitate comparative analyses in this study. The rate of outpatient care (untreated morbidity) was defined as the spells of outpatient care (untreated morbidity) per 1000 of the population exposed to the risk. The source of outpatient care was categorized as public and private facilities.

### Statistical analysis

Bivariate and multivariate logit regression analyses were used to study the socioeconomic differentials in self-reported morbidity (SRM), outpatient care and untreated morbidity. We used horizontal inequity index (HI) to measure the extent of deviation in the use of healthcare for the people in equal need for healthcare irrespective of their income (Wagstaff *et al.* 1991; Van Doorslaer *et al.* 2000; Wagstaff and van Doorslaer 2000). For multivariate and inequity analyses we focused on the individuals who reported being sick in the 15-days reference period. We preferred an indirect method to standardize the use of healthcare for the differences in need because it is computationally straight forward and does not depend on grouped data (Wagstaff and van Doorslaer 2000). We estimated the following probit regression model:

(1) $$y_{i}=P\;\left(\alpha +\sum_{j}\beta_{j}x_{ji}+\;\sum_{k}\gamma_{k}z_{ki}\right)\;+\;\varepsilon_{i}$$

Where, $y_{i}$ was an indicator of healthcare use; i were the individuals and α, $\beta_{j}$ and $\gamma_{k}$ were parameter vectors. The$\;x_{j}$ were need variables for which we adjusted for and $z_{k}$ were non-need variables for which we controlled for to reduce potential bias that would arise if non-need variables correlated to need variables were omitted from the regression (Doorslaer *et al.* 2004; O’Donnell and Wagstaff 2008). We used age (six dummies), gender, reporting of a pre-existing disease, duration of illness and confinement to bed within the reference period to measure the need for healthcare (Supplementary Table A1). Non-need variables such as marital status, social group, education, place of residence, states and MPCE were controlled for. Regression parameter estimates ($\hat{\alpha }$,$\hat{\beta_{j}}$,${\hat{\gamma }}_{k}$), individual values of confounding variables ($x_{ji})$ and sample means of the non-confounding variables (${\overset{-}{z}}_{k}$) were then used to obtain the predicted values of healthcare use (${\hat{y}}_{i}^{x}$):

(2) $${\hat{y}}_{i}^{x}=\;\hat{\alpha }+\;\sum_{j}{\hat{\beta }}_{j}x_{ji}+\;\sum_{k}{\hat{\gamma }}_{k}z_{ki}$$

**Table 1.** Characteristics of spells of ailment reported in 15-days reference period for the population under 60 years and 60 years or more by less and more developed states in India, 1995–96 and 2014

	India		Less developed states		More developed states	
	
Characteristics	1995–96	2014	1995–96	2014	1995–96	2014
	
	Under 60 years					
SRM rate per1000	48.8 (47.9, 49.8)	85.2 (82.9, 87.4)	46.2 (44.9, 47.6)	56.2 (53.6, 58.7)	51.2 (49.8, 52.7)	116.9 (113.1, 120.7)
Healthcare use						
Hospitalization rate per 1000	0.6 (0.5, 0.7)	4.2 (3.9, 4.4)	0.4 (0.3, 0.5)	2.4 (2.1, 2.6)	0.8 (0.6, 1.0)	6.2 (5.7, 6.6)
Outpatient care rate per 1000	41.5 (40.7, 42.3)	66.9 (65.0, 68.8)	38.6 (37.5, 39.7)	42.0 (39.8, 44.1)	44.3 (43.1, 45.5)	94.2 (90.9, 97.5)
Untreated morbidity rate per 1000	6.5 (6.1, 6.8)	14.1 (13.1, 15.0)	7.0 (6.5, 7.5)	11.8 (10.6, 13.1)	5.9 (5.5, 6.4)	16.5 (15.1, 18.0)
Source of outpatient care						
Outpatient care rate in public facilities per 1000	7.2 (6.8, 7.6)	16.3 (15.3, 17.2)	6.3 (5.9, 6.7)	11.9 (10.7, 13.0)	8.1 (7.4, 8.8)	21.1 (19.5, 22.7)
	60 years or more					
SRM rate per 1000	176.3 (168.1, 184.4)	347.3 (332.1, 362.6)	147.2 (137.2, 157.2)	198.5 (180.4, 216.5)	201.2 (188.8, 213.6)	463.0 (439.8, 486.2)
Healthcare use						
Hospitalization rate per 1000	3.6 (2.6, 4.7)	24.0 (22.2, 25.9)	1.3 (0.8, 1.9)	13.6 (11.1, 16.1)	5.6 (3.6, 7.6)	32.1 (29.5, 34.8)
Outpatient care rate per 1000	131.3 (124.4, 138.2)	286.7 (272.9, 300.6)	109.9 (101.3, 118.5)	146.8 (131.6, 162.0)	149.7 (139.1, 160.2)	395.5 (374.0, 417.0)
Untreated morbidity rate per 1000	40.3 (36.4, 44.1)	36.6 (31.0, 42.1)	35.3 (30.5, 40.1)	38.1 (29.1, 47.1)	44.5 (38.7, 50.4)	35.4 (28.4, 42.3)
Source of outpatient care						
Outpatient care rate in public facilities per 1000	25.7 (22.7, 28.7)	77.6 (70.2, 84.9)	20.7 (16.9, 24.5)	51.1 (42.5, 59.7)	30.0 (25.6, 34.5)	98.2 (87.1, 109.3)

Estimates of the indirectly need-standardized use (${\hat{y}}_{i}^{Is}$) was obtained as the difference between actual ($y_{i}$) and need-expected use (${\hat{y}}_{i}^{x}$) plus the overall sample mean ($\overset{-}{y}$):

(3) $${\hat{y}}_{i}^{Is}=y_{i}-{\hat{y}}_{i}^{x}+\overset{-}{y}$$

The distribution of ${\hat{y}}_{i}^{Is}$ across MPCE quintiles was interpreted as the distribution of healthcare use that would be expected to be observed, irrespective of differences in the distribution of the x’s across MPCE quintiles. The concentration curve (CC) which plots the cumulative proportions of population (ranked by MPCE) on the x-axis against the cumulative proportions of healthcare use on y-axis was used to graphically present the inequity. To quantify the magnitude of inequity in healthcare utilization we calculated the concentration index for the need-standardized use (${\hat{y}}_{i}^{Is}$) which was termed as HI (Wagstaff and van Doorslaer 2000). HI was calculated by running the following regression (Kakwani *et al.* 1997):

(4) $$\frac{2\sigma_{R}^{2}}{\bar{y}}{\hat{y}}_{i}^{Is}=\;\alpha +\beta R_{i}+\varepsilon_{i}$$

Where ${\hat{y}}_{i}^{Is}\;$was the need standardized healthcare use, $\bar{y}$ was its mean, $R_{i}$ was the weighted relative fractional rank of the *i*th individual in the consumption distribution, (Ri =1N ∑j=1i-1wj + 12 wi, where $w_{i}$ was the sampling weight w0  =0 and N was the sample size), $\sigma_{R}^{2}$ was the weighted variance of${\;R}_{i}$. The ordinary least square estimate of the slope coefficient gave the estimate of HI (range: −2 to +2). A negative (positive) value implied that the need standardized use of healthcare was disproportionately concentrated among the poor (rich), while a value of zero indicated no inequity (Wagstaff *et al.* 1991). We reported 95% concentration index (CI) for the estimates.

## Results

### Trends in SRM, outpatient care and untreated morbidity

The SRM rate per 1000 in the 15-days reference period increased 1.97 times for the older population, the increase was higher in the more developed compared with the less developed states (2.30 vs 1.35 times) (Table 1). The older population in more developed states had higher SRM rate than the less developed states; this differential was greater in 2014 than in 1995–96 (2.33 vs 1.37 times). Between 1995–96 and 2014, the outpatient care rate increased 2.18 times for the older population. When compared with the less developed states, the older population in more developed states had higher outpatient care rate and also experienced a greater increase (1.34 vs 2.71 times) by 2014. The untreated morbidity rate per 1000 increased marginally in the less developed states (35.3 vs 38.1) but declined in the more developed states (43.4 vs 35.4). The rate of use of public facilities increased 3.02 times for the older population, the increase was higher in the more developed than the less developed states (3.27 vs 2.47 times).

When compared with the younger population, the older population had 3.61 times higher SRM rate in 1995–96 (48.8 vs 176.3 per 1000) and a greater increase by 2014 (1.75 vs 1.97 times). The older population had 3.16 times higher outpatient care rate in 1995–96 (131.3 vs 41.5 per 1000) and a greater increase by 2014 than the younger population (2.18 vs 1.61 times). The older population had 6.20 times higher untreated morbidity rate compared with their younger counterparts in 1995–96; however this differential declined to 2.60 times in 2014. Increase in the use of public facilities for outpatient care was higher for the older population than the younger population (3.02 vs 2.26 times).

### Self-reported morbidity

**Figure 1.**
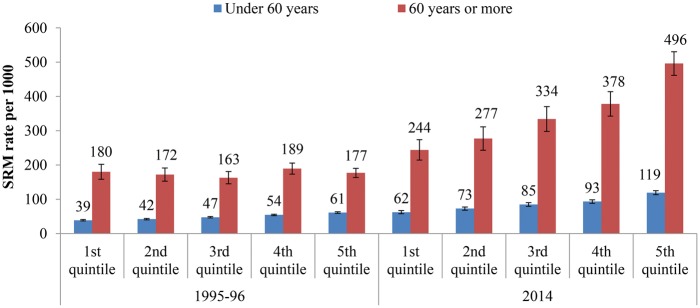
Differentials in SRM rate per 1000 in the 15-days reference period by MPCE quintiles for the population under 60 years and 60 years or more in India, 1995–96 and 2014

A clear economic gradient with richer quintiles reporting higher morbidity was observed for the older population in 2014 and for the younger population in both years. In both the years, SRM rate was higher for the older population in each consumption quintile than the younger population (range: 2.89–4.64 times). The increase in SRM rate was highest in the richest quintile; more so for the older population than the population under 60 years (2.80 vs 1.95 times) (Figure 1).

The top four most frequently reported ailments for the older population were non-communicable diseases. Musculoskeletal problem was the most frequently neglected disease for the older population. Among older population, only 2 out of 10 most frequently reported aliments were communicable diseases and 7 out of 10 most frequently neglected ailments were NCDs. Fevers of all types and acute upper respiratory infections were the top two most frequently reported and most frequently neglected ailments for the population under 60 years (Table 2).

**Table 2.** Profile of the incidence and neglect of top10 SRM in 15-days reference period for the population under 60 years and 60 years or more in India, 2014

Most frequently reported morbidity	Under 60 years	Most frequently reported morbidity	60 years or more	Most frequently reported morbidity that remained untreated	Under 60 years	Most frequently reported morbidity that remained untreated	60 years or more
All fevers^a^	22.2	Hypertension	20.9	All fevers^a^	22.2	Joint or bone disease^c^	19.0
Acute upper respiratory infections^b^	9.6	Diabetes	16.5	Acute upper respiratory infections^b^	18.6	All fevers^a^	15.6
Diabetes	7.5	Joint or bone disease^c^	13.6	Headache	7.1	Back or body aches	9.3
Hypertension	6.0	Bronchial asthma^d^	6.5	Joint or bone disease^c^	6.6	Gastric/peptic ulcer	6.6
Joint or bone disease^c^	5.9	All fevers^a^	5.5	Cough with sputum with or without fever not diagnosed as tuberculosis	5.5	Hypertension	6.0
Gastric/peptic ulcer	5.4	Heart disease	5.5	Gastric/peptic ulcer	5.3	Acute upper respiratory infections^b^	5.3
Cough with sputum with or without fever not diagnosed as tuberculosis	3.0	Back or body aches	4.0	Diarrhoea/dysentry	4.7	Diabetes	5.0
Back or body aches	2.9	Gastric/peptic ulcer	3.8	Back or body aches	4.3	Bronchial asthma^d^	4.3
Bronchial asthma^d^	2.9	Symptoms not fitting into any categories	2.6	Fever due to diptheria/whooping cough	2.5	Diarrhoea/dysentry	3.6
Diarrhoea/dysentry	2.7	Acute upper respiratory infections^b^	2.5	Skin infection and other skin diseases	2.0	Skin infection and other skin diseases	2.9

a Fever due to malaria, typhoid and fever of unknown origin, all specific fevers that do not have a confirmed diagnosis;

b Cold, runny nose, sore throat with cough, allergic colds included;

c Pain or swelling in any joints, or swelling or pus from the bones;

d Recurrent episode of wheezing and breathlessness with or without cough over long periods or known asthma.

### Horizontal inequity in outpatient care and untreated morbidity

**Figure 2.**
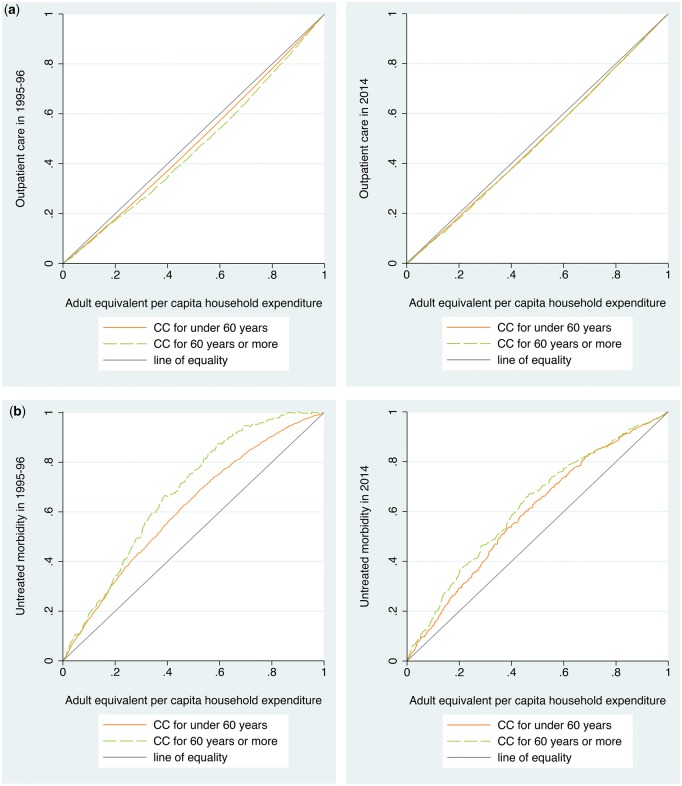

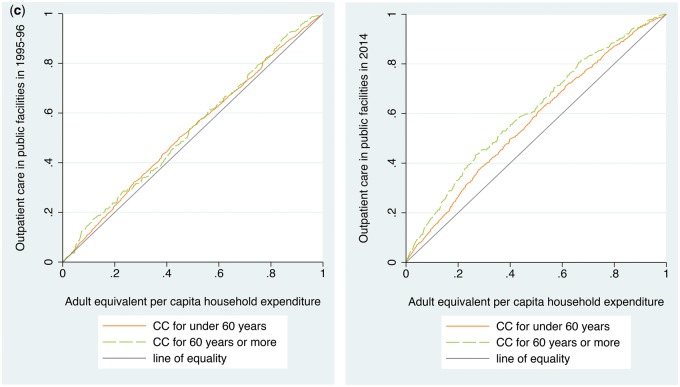
(a) Concentration curves for the use of outpatient care for the population under 60 years and 60 years or more in India, 1995–96 and 2014. (b) Concentration curves for untreated morbidity for the population under 60 years and 60 years or more in India, 1995–96 and 2014. (c) Concentration curves for the use of public facilities for outpatient care for the population under 60 years and 60 years or more in India, 1995–96 and 2014

In each consumption quintile the older population had higher outpatient care rate than the younger population at both time points (range: 2.83–4.32 times) (Table 3). Between 1995–96 and 2014, outpatient care rates increased most for the richest quintile; this increase was higher for the older population than the younger population (2.73 vs 1.79 times). The CC indicated that for equal need the use of outpatient care was higher for the richer quintiles at both time points irrespective of age groups (Figure 2a). The corresponding positive HI values confirmed the pro-rich bias in outpatient care. The older population had higher pro-rich inequity in outpatient care than the younger population in 1995–96 (HI: 0.085; 95% CI: 0.066, 0.103 vs 0.039; 0.034, 0.043). The pro-rich inequity in outpatient care declined for the older population (0.085; 0.066, 0.103 vs 0.027; 0.015, 0.039) but not for the younger population (0.039; 0.034, 0.043 vs 0.030; 0.022, 0.037) which resulted into similar levels of inequity in both age groups by 2014.

**Table 3.** Outpatient care, untreated morbidity and the use of public facilities for outpatient care for the population under 60 years and 60 years or more by MPCE quintiles in India, 1995–96 and 2014

MPCE quintiles^a^	Under 60 years					
	Outpatient care rate per 1000^b^		Untreated morbidity rate per 1000^b^		Outpatient care rate in public facilities per 1000^b^	
	1995–96	2014	1995–96	2014	1995–96	2014
Poorest	29.6 (27.7, 31.6)	45.0 (41.1, 48.9)	8.4 (7.4, 9.3)	15.1 (12.8, 17.4)	5.6 (4.9, 6.3)	14.3 (12.3, 16.2)
Poor	33.8 (31.9, 35.8)	54.3 (50.3, 58.2)	7.3 (6.5, 8.2)	15.8 (13.4, 18.1)	6.7 (5.6, 7.7)	16.5 (14.3, 18.7)
Middle	39.8 (37.8, 41.8)	66.5 (61.6, 71.3)	6.9 (6.1, 7.7)	14.3 (12.2, 16.4)	7.0 (6.1, 8.0)	16.6 (14.4, 18.9)
Rich	47.7 (45.7, 49.7)	74.9 (70.1, 79.6)	5.8 (5.0, 6.5)	13.3 (11.2, 15.3)	7.5 (6.8, 8.1)	16.3 (14.1, 18.4)
Richest	56.3 (53.9, 58.6)	100.6 (95.0, 106.1)	3.9 (3.4, 4.5)	11.4 (9.5, 13.4)	9.3 (8.1, 10.5)	18.2 (15.8, 20.5)
HI (95% CI)	0.039 (0.034, 0.043)	0.030 (0.022, 0.037)	−0.240 (−0.268, −0.212)	−0.176 (−0.211, −0.141)	−0.056 (−0.086, −0.026)	**−**0.145 (−0.175, −0.115)
	60 years or more					
Poorest	115.1 (96.3, 133.9)	176.5 (151.5, 201.6)	60.7 (49.9, 71.6)	56.9 (40.8, 72.9)	26.1 (19.0, 33.1)	78.9 (62.1, 95.6)
Poor	110.3 (94.5, 126.1)	223.7 (192.7, 254.7)	56.7 (46.1, 67.4)	38.4 (24.2, 52.7)	19.2 (14.4, 24.1)	81.3 (61.3, 101.3)
Middle	118.6 (103.7, 133.5)	269.3 (235.6, 302.9)	39.3 (29.9, 48.7)	40.9 (28.3, 53.6)	25.9 (18.6, 33.1)	64.8 (50.2, 79.3)
Rich	156.2 (141.3, 171.2)	323.8 (289.5, 358.1)	27.2 (22.0, 32.4)	25.2 (16.4, 34.0)	30.5 (23.0, 38.0)	88.1 (70.9, 105.3)
Richest	159.0 (146.2, 171.8)	434.7 (401.5, 467.9)	14.5 (10.6, 18.4)	20.5 (12.7, 28.2)	27.1 (20.7, 33.6)	74.8 (61.3, 88.3)
HI (95% CI)	0.085 (0.066, 0.103)	0.027 (0.015, 0.039)	−0.268 (−0.312, −0.223)	−0.320 (−0.391, −0.249)	−0.065 (−0.129, −0.001)	−0.189 (−0.234, −0.145)

a Monthly per capita consumption expenditure quintiles;

b Reference period is 15-days prior to the date of survey.

The poorest older population had 4.19 times higher untreated morbidity rate than the richest in 1995–96 which declined to 2.78 times in 2014. The gap between the poorest and the richest in untreated morbidity was higher for the older population than the population under 60 years (4.19 vs 2.12 times). The CC showed that the untreated morbidity was disproportionately concentrated among the poor; more so for the older population than the population under 60 years (Figure 2b). Even after adjusting for the differences in need, untreated morbidity was reported more by the poor as indicated by the negative value of HI. The magnitude of inequity in untreated morbidity was higher for the older population (−0.320; −0.391, −0.249) than the younger population (−0.176; −0.211, −0.141) in 2014 (Table 3).

Between 1995–96 and 2014, the use of public facilities for outpatient care among the older population increased most in the poorest (3.03 times) and the poor quintiles (4.23 times). The younger population showed similar pattern but of lower magnitude (Table 3). The CC for the use of public facilities was above the line of equality which indicated that the use was disproportionately higher among the poor individuals, particularly in the latter time point (Figure 2c). The pro-poor inequity in the use of public facilities increased between 1995–96 and 2014 for the older population (−0.065; −0.129, −0.001 vs −0.189; −0.234, −0.145) and the population under 60 years (−0.056; −0.086, −0.026 vs −0.145; −0.175, −0.115).

### Determinants of outpatient care and untreated morbidity

The poorest had significantly lower adjusted odds of using outpatient care than the richest at both the time points. The poorest older population were 80% (95% CI: 0.13–0.31) less likely to use outpatient care than the richest in 1995–96; this gap declined by 2014 where the poorest were only 36% (95% CI: 0.43, 0.94) less likely to use outpatient care (Table 4). In 1995–96, the older population suffering from a pre-existing disease and with duration of illness >11 days were 48% (95% CI: 0.36, 0.75) and 41% (95% CI: 0.52, 0.84) less likely to use outpatient care, respectively. Older population confined to bed due to illness were 2.42 (95% CI: 1.80, 3.26) times more likely to use outpatient care in 1995–96; however by 2014 they had 31% (95% CI: 0.51, 0.95) lower odds of treatment as an outpatient.

**Table 4.** Determinants of outpatient care, untreated morbidity and the use of public facilities for outpatient care for the population 60 years or more in India, 1995–96 and 2014

Background characteristics	60 years or more					
	Outpatient care		Untreated morbidity		Use of public facilities for outpatient care	
	1995–96	2014	1995–96	2014	1995–96	2014
Age (Ref. 60–69 years)						
70–79	0.80 (0.61, 1.05)	0.88 (0.69, 1.12)	1.25 (0.95, 1.65)	0.98 (0.67, 1.42)	1.30 (0.97, 1.76)	0.90 (0.70, 1.17)
80 years or more	0.76 (0.52, 1.10)	0.62 (0.46, 0.85)	1.42 (0.97, 2.08)	1.59 (1.02, 2.47)	0.93 (0.59, 1.47)	1.07 (0.73, 1.57)
Gender (Ref. = Male)						
Female	0.90 (0.70, 1.15)	1.01 (0.80, 1.29)	1.16 (0.90, 1.50)	1.18 (0.83, 1.69)	1.05 (0.77, 1.44)	1.05 (0.81, 1.35)
Duration of illness (Ref. = <11 days)						
12 days or more	0.59 (0.42, 0.84)	1.25 (0.77, 2.03)	1.59 (1.12, 2.26)	0.45 (0.25, 0.79)	1.01 (0.68, 1.50)	1.30 (0.73, 2.31)
Whether confined to bed (Ref. = No)						
Yes	2.42 (1.80, 3.26)	0.69 (0.51, 0.95)	0.41 (0.30, 0.56)	0.53 (0.32, 0.89)	0.90 (0.69, 1.19)	0.90 (0.61, 1.34)
Whether suffering from a pre-existing disease (Ref. = No)						
Yes	0.52 (0.36, 0.75)	1.49 (0.89, 2.49)	1.81 (1.24, 2.65)	0.59 (0.32, 1.06)	1.08 (0.72, 1.62)	0.98 (0.54, 1.78)
MPCE quintiles (Ref. = Richest)						
Poorest	0.20 (0.13, 0.31)	0.64 (0.43, 0.94)	5.20 (3.34, 8.10)	3.39 (1.85, 6.20)	1.55 (0.89, 2.68)	2.90 (1.90, 4.43)
Poor	0.21 (0.14, 0.30)	0.81 (0.54, 1.21)	4.99 (3.35, 7.42)	2.56 (1.34, 4.87)	1.06 (0.65, 1.71)	2.22 (1.51, 3.27)
Middle	0.27 (0.18, 0.39)	0.70 (0.51, 0.96)	3.62 (2.41, 5.44)	2.57 (1.49, 4.43)	1.39 (0.87, 2.21)	1.29 (0.91, 1.84)
Rich	0.58 (0.40, 0.82)	0.96 (0.72, 1.28)	1.65 (1.13, 2.41)	1.34 (0.78, 2.30)	1.21 (0.81, 1.79)	1.58 (1.15, 2.17)
Marital status (Ref. = Currently married)					
Single	0.68 (0.52, 0.89)	1.15 (0.91, 1.47)	1.47 (1.12, 1.92)	0.94 (0.67, 1.31)	0.97 (0.71, 1.32)	1.09 (0.84, 1.42)
Caste (Ref. = Non SC/STs)						
SC/STs	1.11 (0.82, 1.49)	0.65 (0.48, 0.89)	0.96 (0.71, 1.29)	1.90 (1.28, 2.83)	1.29 (0.92, 1.81)	1.46 (1.09, 1.95)
Place of residence (Ref. = Urban)						
Rural	0.67 (0.52, 0.85)	0.85 (0.68, 1.06)	1.57 (1.21, 2.04)	1.28 (0.91, 1.81)	1.14 (0.87, 1.49)	1.14 (0.89, 1.46)
Education (Ref. = Literate)						
Illiterate	0.82 (0.64, 1.04)	1.05 (0.79, 1.39)	1.23 (0.96, 1.57)	0.89 (0.58, 1.37)	0.85 (0.63, 1.14)	1.21 (0.93, 1.56)
States (Ref. = More developed states)						
Less developed states	0.91 (0.71, 1.17)	0.67 (0.53, 0.86)	1.22 (0.95, 1.57)	1.61 (1.14, 2.28)	0.93 (0.70, 1.22)	1.19 (0.90, 1.58)
Constant	37.42 (23.44, 59.76)	4.95 (3.30, 7.43)	0.02 (0.01, 0.04)	0.10 (0.06, 0.19)	0.18 (0.12, 0.26)	0.14 (0.09, 0.22)
***n***	6010	10 236	6010	10 236	4421	7465

When compared with the richest, the poorest older population were 5.20 (95% CI: 3.34, 8.10) times and 3.39 (95% CI: 1.85, 6.20) times more likely to remain untreated in an event of illness in 1995–96 and 2014, respectively. Older population confined to bed due to illness were significantly less likely to remain untreated in 1995–96 and 2014. Older population suffering from a pre-existing disease and with duration of illness >11 days were 1.59 (95% CI: 1.12, 2.36) times and 1.81 (95% CI: 1.24, 2.65) times more likely to remain untreated in 1995–96. The older population residing in less developed states were 1.61 times significantly more likely to remain untreated in 2014.

After adjusting for the confounders, MPCE quintiles was not a significant predictor of the use of public facilities for outpatient care for the older population in 1995–96. However, in 2014, the poorest older population were 2.90 (95% CI: 1.90–4.43) times more likely to use public facilities (Table 4). The older population belonging to SC/STs had 1.46 times significantly higher odds of using public facilities in 2014. The adjusted association of MPCE quintiles with the three outcome variables of healthcare utilization followed similar pattern for the population under 60 years; however the magnitude was different (Supplementary Table A2).

### Barriers to healthcare utilization

An ailment not being considered serious by the respondent was the most important reason for not seeking treatment on medical advice followed by ‘other’ reasons both for the poor and the non-poor older population. A financial reason for not seeking treatment was 9.9 percentage points higher for the poor than the non-poor older population. Also the unavailability of a medical facility in the neighbourhood was reported more by the poor than the non-poor older population. Even for the younger population, an ailment not considered serious was the single most important reason reported both by the poor and the non-poor; with levels higher than the older population (Table 5).

**Table 5.** Reasons for not seeking treatment on medical advice and not using public facilities for outpatient care by the non-poor and the poor population under 60 years and 60 years or more in India, 2014

Reasons for not seeking treatment on medical advice	Under 60 years			60 years or more		
	Non-poor	Poor	Total	Non-poor	Poor	Total
No medical facility available in the neighbourhood	9.5	13.2	12.0	7.1	12.0	10.8
Facility of satisfactory quality not available	1.6	3.5	2.9	9.3	3.6	5.0
Facility of satisfactory quality too expensive	2.3	5.3	4.4	5.9	15.8	13.3
Facility of satisfactory quality involves long waiting	2.6	3.5	3.2	4.6	1.5	2.3
Ailment not considered serious	71.3	60.7	64.2	45.8	42.3	43.2
Others	12.7	13.8	13.5	27.3	24.7	25.4
Reasons for not using public facilities for outpatient care						
Required specific service not available	8.4	12.2	10.6	6.3	10.3	8.4
Facility available but quality not satisfactory	42.0	40.9	41.4	45.6	45.2	45.4
Quality satisfactory but facility too far	10.7	13.8	12.5	6.7	13.1	10.0
Facility of satisfactory quality but involves long waiting	29.6	24.9	26.9	33.8	26.1	29.8
Financial constraint	0.2	0.7	0.5	0.1	1.0	0.6
Others	9.1	7.6	8.3	7.5	4.4	5.9

Unsatisfactory quality was the dominant reason for not using public facilities reported equally by the poor and non-poor older population in 2014. A long waiting time and ‘other reasons’ for not using public facilities was higher for the non-poor while the unavailability of a public facility was reported more by the poor older population. The gap between poor and non-poor in reporting ‘facility too far’, a long waiting time and ‘other reasons’ for not using public facilities was higher for the older population than the younger population (Table 5).

## Discussion

Our findings show that the economic status is a strong independent determinant of healthcare use in India. The horizontal inequity in the use of outpatient care favoured the rich while the poor had more untreated morbidity. There was however, a difference in the degree to which horizontal inequities in healthcare use occurred in the two age groups over the two decades. Three salient findings related to inequity emerge from this study. First, the pro-rich inequity in the use of outpatient care among the older population declined between 1995–96 and 2014. Second, the untreated morbidity was disproportionately higher among the poor; more so for the older population and the inequity increased over the last two decades. Third, the proportion of poor people using public facilities for outpatient care was higher than the rich in 1995–96, and this gap increased over the next 20 years.

Socioeconomic inequality in the use of healthcare with the wealthier population group having a higher probability of using healthcare when needed is a persistent phenomenon in low- and middle-income countries (Makinen *et al.* 2000; Ahmed *et al.* 2005; Bourne 2009; Peltzer *et al.* 2014). Even in countries with universal health coverage the use of healthcare was found to be disproportionately concentrated among the richer groups after adjusting for the differences in healthcare needs (Dunlop *et al.* 2000; Shin and Kim 2010). Equitable access to preventive and curative care will help in averting deaths and diseases leading to better health outcomes. In this study, we focused on the inequity in outpatient care because it is the entry point for most people in the healthcare system and can have an effect on the use of other services as well (Peters *et al.* 2001; Malhotra and Do 2012; Kavosi *et al.* 2015). The pro-rich inequity in the use of outpatient care found in our study is consistent with the evidence from other studies in India and China (Ghosh 2014a; Zhang *et al.* 2015). On a positive note, we found that the pro-rich inequity in the use of outpatient care declined significantly for the older population over the past two decades. Increase in the government funded insurance schemes, improved physical and financial access to public healthcare services and the increased awareness about the treatable medical conditions might have contributed to the increase in outpatient care among the poor older population. Highly subsidized healthcare, high insurance coverage and low cost of healthcare are important means to achieve equitable access to outpatient care for all (Hidayat *et al.* 2004; Kavosi *et al.* 2015).

Both the levels of untreated morbidity and the difference in untreated morbidity rate between the richest and the poorest quintiles was substantially higher for the older population at both time points. Untreated morbidity is common among the older population because they generally associate their illness with the ageing process and neglect medical treatment (Biswas *et al.* 2006; Mukherjee and Karmakar 2008). They usually start with self-medication and seek care from qualified medical professionals only when health conditions deteriorate (Pang *et al.* 2003; Waweru *et al.* 2003). A previous study comparing 23 low- and middle-income countries found that the older population suffering from disability and chronic disease tend to avoid healthcare (Abegunde *et al.* 2007). Equity in healthcare has greater importance for the older population because of their greater need for medical care and consequently higher demand for health services (Wallace and Gutiérrez 2005).

We found a high level of reporting of morbidity among the older population indicating that they have a greater need for healthcare. The older population most frequently reported non-communicable diseases and showed a tendency to forgo medical treatment when the reported morbidity was perceived to be age-related. Evidence suggests that ignoring minor symptoms or early signs of chronic diseases might lead to severe consequences that would require more medical treatment and involve higher cost (Murata *et al.* 2010). Our observation is important in light of the inadequate provision of geriatric healthcare services in India. Only recently India has initiated the National Programme for the Healthcare of Elderly to promote active and healthy ageing. The policy targets a range of services including diagnosis and management of geriatric medical problems to deal with the increasing disease burden among the older population (Ministry of Health and Family Welfare 2011b).

The SRM rate increased nearly 2-fold over the past two decades in India. This can partly be due to the increase in disease burden of the country given the population ageing and higher morbidity prevalence at older ages. Data from the Global Burden of Disease Study show that total disease burden measured as the disability adjusted life years lost in India has increased for the older population from 67 million in 1990 to 110 million in 2013 (Institute for Health Metrics and Evaluation (IHME) 2015). Another reason could be the enhanced perception of morbidity as captured by the self-reported responses of being ill in the reference period. However, in the absence of any objective measure of health or a vignette schedule in the NSSO surveys it would be difficult to judge how much of the increase in the level of reported illness can be attributed to real increase in burden of disease and how much to the enhanced subjective perception of illness.

We found substantial variation in SRM rate by economic status and states in India. Residing in the more developed states and having higher socioeconomic position was found to be associated with higher reporting of morbidity. This could be attributed to ‘perception bias’; a tendency among the deprived to report less ill-health and underestimate their health problems (Sen 2002). The individuals in the more developed states have improved provision of health services and are therefore in a better position to perceive and report health problems than their counterparts in the less developed states. Economically disadvantaged people lack awareness about treatable medical conditions and often do not consider themselves ill due to the high cost of healthcare and accessibility issues (Sen 1993; Dilip 2002; Prinja *et al.* 2015). The differential rates of epidemiological transition between different socioeconomic strata could be another explanation for the high prevalence of SRM among the rich people (Prinja *et al.* 2012a, 2015).

We found that the perceived non-serious nature of the ailment was the most important reason for not seeking medical treatment irrespective of economic status. This confirms that apart from supply and economic constraints, the demand for healthcare is also affected by the individual’s perception of their medical conditions. In contrast to this, a previous study in India using data from the NSSO 2004 showed that in the poorer consumption quintiles financial reasons dominated, while in the richer quintiles self-perception of illness was the prominent reason (Mukherjee and Karmakar 2008). Evidence from India also suggests that financial barriers hinder the healthcare use of the poor, particularly in times of health sector reforms (Ghosh 2014b). However, our finding suggested that the high cost of healthcare remains a persistent barrier to medical treatment only for the poor older population. Economic factors are more important in determining the healthcare of the older population because of their higher economic dependency and poverty as a result of a lack of a regular source of income (Ministry of Social Justice and Empowerment 1999; Srivastava and Mohanty 2012). The ‘other’ reason for not seeking medical care was high for the older population, indicating that they have greater preference to seek informal care when ill.

Even though public healthcare in India has the provision of free or low-cost services, the utilization of public facilities was found to be low due to the perceived poor quality of services. Higher use of private facilities for healthcare has been reported by other studies in India (Levesque *et al.* 2006; Selvaraj and Karan 2009; Malhotra and Do 2012; Brinda *et al.* 2015; Goeppel *et al.* 2016). The heavy reliance on the private sector indicates that the public sector has not kept up with the growing demand for healthcare. We found that the use of public facilities was disproportionately higher for the poor than the rich and the gap increased over the past two decades. Another study in India using the NSSO 1995–96 data also showed that the outpatient treatment in public facilities was distributed in favour of the population living below poverty line (Mahal *et al.* 2001a). Increased use of outpatient care from public facilities by the poor might be due to their decreased ability to bear the increasingly high cost of outpatient care in the private sector. Lack of resources and the high cost involved in private facilities might have forced the poor to revert to public facilities. Evidence suggests that the publically financed health services in India represent the best way for providing critical services for the poor (Mahal *et al.* 2001b). Therefore, investments to improve the quality of services in public facilities would help in securing affordable health services in India, particularly for those who cannot afford the expensive private healthcare (Levesque *et al.* 2006).

There are some limitations of this study which should be taken into account while interpreting our results. First, the use of self-reported data on morbidity to adjust for the need for healthcare might suffer from health perception bias. Second, we couldn’t make causal inference between economic status and healthcare use. Third, no adjustment could be made for the differences in quality of healthcare while studying inequity in utilization. Fourth, we could use only individual level determinants of healthcare use. Other factors like culture, community and health system reforms are likely to affect the use of healthcare which could not be included due to the lack of such information in these national sample surveys. In spite of the limitations, this study provides large scale evidence on how the inequity in healthcare use has changed over the last two decades in India that could inform health policy.

In conclusion, this study provides evidence of a higher burden of SRM, greater use of outpatient care, higher level of untreated morbidity and greater inequities in healthcare use for the older population compared with rest of the population in India. Policy initiatives aiming to reduce these inequities in healthcare use should focus on increasing public investment in health, providing insurance coverage for outpatient care and making better provisions for geriatric healthcare in India.

## Ethical statement

The study is based on secondary data from the National Sample Surveys with no identifiable information on the survey participants. Exemption from ethics approval for analysis of the National Sample Surveys data was obtained from the institutional ethics committees of the Public Health Foundation of India and the London School of Hygiene and Tropical Medicine.

## Supplementary data

Supplementary data are available at *HEAPOL* online.

## Funding

This work was supported by a Wellcome Trust Capacity Strengthening Strategic Award to the Public Health Foundation of India and a consortium of UK universities. It is part of Anamika Pandey’s PhD for which she is registered at the London School of Hygiene and Tropical Medicine.

*Conflict of interest statement*. None declared.

## Supplementary Material

Appendix Tables
