# Supplementary material for: Horizontal inequity in outpatient care use and untreated morbidity: evidence from nationwide surveys in India between 1995 and 2014
Source: Health Policy Plan. 2017 Apr 17;32(7):969–79. doi: 10.1093/heapol/czx016 (PMC5886081; doi:10.1093/heapol/czx016)
Supplement: Appendix Tables [file appendix_tables_czx016.docx]

Appendix Table 1. Distribution of need variables by MPCE* quintiles for the population under 60 years and 60 years or more in India, 1995-96 and 2014.

| Need variables | Under 60 years | | | | | | | | | |
| --- | --- | --- | --- | --- | --- | --- | --- | --- | --- | --- |
|  | 1995-96 | | | | | 2014 | | | | |
| **Age (years)** | Poorest | Poor | Middle | Rich | Richest | Poorest | Poor | Middle | Rich | Richest |
| 0-14 | 35.3 | 40.6 | 42.2 | 39.7 | 36.0 | 28.7 | 32.6 | 26.9 | 27.0 | 19.2 |
| 15-29 | 23.1 | 20.0 | 18.6 | 20.4 | 20.5 | 18.0 | 18.0 | 17.2 | 15.9 | 13.6 |
| 30-44 | 20.5 | 20.7 | 20.8 | 21.8 | 23.5 | 22.1 | 23.4 | 25.9 | 26.1 | 24.9 |
| 45-59 | 21.1 | 18.8 | 18.4 | 18.1 | 20.0 | 31.2 | 26.0 | 29.9 | 31.1 | 42.3 |
| **Gender** |  |  |  |  |  |  |  |  |  |  |
| Male | 50.1 | 48.6 | 48.1 | 49.3 | 50.2 | 45.5 | 44.0 | 43.8 | 44.9 | 43.6 |
| Female | 49.9 | 51.4 | 51.9 | 50.7 | 49.8 | 54.5 | 56.0 | 56.2 | 55.1 | 56.5 |
| **Number of days ill in the 15 days reference period** |  |  |  |  |  |  |  |  |  |  |
| 1-11 days | 75.6 | 76.1 | 72.8 | 70.1 | 64.6 | 62.6 | 59.1 | 51.1 | 51.0 | 38.9 |
| 12-15 days | 24.4 | 23.9 | 27.2 | 29.9 | 35.4 | 37.4 | 40.9 | 49.0 | 49.0 | 61.2 |
| **Whether confined to bed in the 15 days reference period** |  |  |  |  |  |  |  |  |  |  |
| No | 60.2 | 63.8 | 64.3 | 66.9 | 68.4 | 78.5 | 80.9 | 86.1 | 84.9 | 90.4 |
| Yes | 39.8 | 36.2 | 35.8 | 33.1 | 31.6 | 21.5 | 19.1 | 13.9 | 15.2 | 9.7 |
| **Whether having a pre-existing disease** | |  |  |  |  |  |  |  |  |  |
| No | 64.4 | 65.3 | 61.8 | 62.1 | 54.5 | 53.7 | 53.0 | 46.8 | 45.9 | 35.5 |
| Yes | 35.6 | 34.7 | 38.2 | 37.9 | 45.5 | 46.3 | 47.1 | 53.2 | 54.1 | 64.5 |
| Need variables | 60 years or more | | | | | | | | | |
|  | 1995-96 | | | | | 2014 | | | | |
| **Age (years)** | Poorest | Poor | Middle | Rich | Richest | Poorest | Poor | Middle | Rich | Richest |
| 60-69 | 61.0 | 56.4 | 59.0 | 53.3 | 58.3 | 58.5 | 57.2 | 59.9 | 63.0 | 63.1 |
| 70-79 | 28.5 | 32.8 | 28.8 | 31.9 | 30.8 | 27.5 | 34.9 | 31.9 | 27.6 | 27.2 |
| 80+ | 10.5 | 10.8 | 12.2 | 14.9 | 10.9 | 14.0 | 7.9 | 8.1 | 9.4 | 9.8 |
| **Gender** |  |  |  |  |  |  |  |  |  |  |
| Male | 50.1 | 49.5 | 48.8 | 52.7 | 49.9 | 45.2 | 47.3 | 47.7 | 45.9 | 49.5 |
| Female | 49.9 | 50.5 | 51.2 | 47.3 | 50.2 | 54.9 | 52.7 | 52.3 | 54.1 | 50.6 |
| **Number of days ill in the 15 days reference period** |  |  |  |  |  |  |  |  |  |  |
| 1-11 days | 43.3 | 39.4 | 44.0 | 33.3 | 24.9 | 33.1 | 19.8 | 15.2 | 12.5 | 9.7 |
| 12-15 days | 56.7 | 60.6 | 56.0 | 66.7 | 75.1 | 66.9 | 80.2 | 84.8 | 87.5 | 90.4 |
| **Whether confined to bed in the 15 days reference period** |  |  |  |  |  |  |  |  |  |  |
| No | 72.0 | 73.6 | 63.6 | 70.0 | 71.8 | 83.8 | 85.5 | 89.0 | 91.6 | 93.0 |
| Yes | 28.0 | 26.5 | 36.4 | 30.0 | 28.2 | 16.2 | 14.5 | 11.0 | 8.4 | 7.0 |
| **Whether having a pre-existing disease** | |  |  |  |  |  |  |  |  |  |
| No | 30.4 | 34.1 | 37.9 | 27.6 | 21.2 | 26.6 | 15.8 | 11.9 | 10.7 | 8.7 |
| Yes | 69.6 | 66.0 | 62.1 | 72.4 | 78.8 | 73.4 | 84.2 | 88.1 | 89.3 | 91.3 |

* Monthly per-capita consumption expenditure.

Appendix Table 2. Determinants of outpatient care, untreated morbidity and the use of public facilities for outpatient care for the population under 60 years in India, 1995-96 and 2014.

| Background characteristics | Under 60 years | | | | | |
| --- | --- | --- | --- | --- | --- | --- |
|  | Outpatient care | | Untreated morbidity | | Use of public facilities for outpatient care | |
|  | 1995-96 | 2014 | 1995-96 | 2014 | 1995-96 | 2014 |
| **Age (Ref. = 0-14 years)** |  |  |  |  |  |  |
| 15 - 29 | 0.84 (0.70, 1.01) | 0.64 (0.51, 0.81) | 1.18 (0.97, 1.43) | 1.50 (1.14, 1.97) | 1.09 (0.90, 1.32) | 1.16 (0.89, 1.52) |
| 30 - 44 | 0.77 (0.61, 0.97) | 0.64 (0.50, 0.83) | 1.30 (1.02, 1.66) | 1.89 (1.41, 2.54) | 1.27 (1.01, 1.60) | 1.33 (0.99, 1.79) |
| 45 - 59 | 0.76 (0.61, 0.94) | 0.84 (0.66, 1.08) | 1.32 (1.06, 1.64) | 1.46 (1.10, 1.95) | 1.43 (1.16, 1.77) | 2.02 (1.53, 2.66) |
| **Gender (Ref. = Male)** |  |  |  |  |  |  |
| Female | 0.89 (0.79, 1.00) | 1.12 (0.98, 1.27) | 1.12 (0.99, 1.26) | 0.90 (0.77, 1.06) | 1.00 (0.88, 1.13) | 1.16 (1.00, 1.34) |
| **Duration of illness (Ref. = less than 11 days)** |  |  |  |  |  |  |
| 12 days or more | 0.81 (0.69, 0.95) | 0.95 (0.76, 1.20) | 1.10 (0.93, 1.29) | 0.64 (0.48, 0.84) | 0.99 (0.85, 1.14) | 1.06 (0.82, 1.37) |
| **Whether confined to bed (Ref. = No)** |  |  |  |  |  |  |
| Yes | 2.21 (1.93, 2.54) | 1.03 (0.86, 1.23) | 0.43 (0.37, 0.50) | 0.57 (0.45, 0.72) | 1.10 (0.96, 1.26) | 1.01 (0.82, 1.23) |
| **Whether suffering from a pre-existing disease (Ref. = No)** | |  |  |  |  |  |
| Yes | 1.12 (0.97, 1.29) | 1.48 (1.19, 1.84) | 0.86 (0.74, 0.99) | 0.57 (0.44, 0.73) | 1.01 (0.88, 1.16) | 1.04 (0.81, 1.33) |
| **MPCE quintiles (Ref. = Richest)** |  |  |  |  |  |  |
| Poorest | 0.30 (0.24, 0.37) | 0.65 (0.52, 0.81) | 3.40 (2.74, 4.23) | 2.15 (1.64, 2.81) | 1.34 (1.06, 1.71) | 1.74 (1.34, 2.26) |
| Poor | 0.38 (0.31, 0.47) | 0.69 (0.56, 0.85) | 2.55 (2.07, 3.15) | 1.98 (1.53, 2.55) | 1.33 (1.05, 1.69) | 1.79 (1.41, 2.27) |
| Middle | 0.49 (0.40, 0.60) | 0.80 (0.66, 0.97) | 2.14 (1.74, 2.62) | 1.55 (1.22, 1.98) | 1.14 (0.92, 1.43) | 1.43 (1.13, 1.80) |
| Rich | 0.67 (0.55, 0.81) | 0.86 (0.71, 1.03) | 1.52 (1.24, 1.87) | 1.36 (1.06, 1.74) | 0.97 (0.80, 1.17) | 1.22 (0.98, 1.51) |
| **Marital status (Ref. = Currently married)** |  |  |  |  |  |  |
| Single | 0.73 (0.61, 0.88) | 0.96 (0.80, 1.15) | 1.40 (1.16, 1.69) | 1.24 (1.00, 1.53) | 1.27 (1.07, 1.50) | 1.39 (1.13, 1.72) |
| **Caste (Ref. = Non SC/STs)** |  |  |  |  |  |  |
| SC/STs | 1.11 (0.97, 1.26) | 0.89 (0.77, 1.03) | 0.93 (0.82, 1.07) | 1.06 (0.89, 1.26) | 1.28 (1.10, 1.48) | 1.65 (1.40, 1.94) |
| **Place of residence (Ref. = Urban)** |  |  |  |  |  |  |
| Rural | 0.70 (0.61, 0.79) | 0.83 (0.73, 0.95) | 1.49 (1.30, 1.70) | 1.22 (1.04, 1.43) | 0.99 (0.86, 1.14) | 1.17 (1.00, 1.36) |
| **Education (Ref. = Literate)** |  |  |  |  |  |  |
| Illiterate | 0.90 (0.80, 1.02) | 0.92 (0.79, 1.07) | 1.14 (1.00, 1.29) | 1.01 (0.84, 1.21) | 0.82 (0.72, 0.94) | 0.81 (0.68, 0.96) |
| **States (Ref. = More developed states)** |  |  |  |  |  |  |
| Less developed states | 0.82 (0.73, 0.92) | 0.83 (0.72, 0.96) | 1.30 (1.15, 1.47) | 1.30 (1.10, 1.53) | 0.86 (0.76, 0.98) | 1.29 (1.10, 1.52) |
| **Constant** | 20.18 (15.42, 26.41) | 5.96 (4.55, 7.80) | 0.04 (0.03, 0.06) | 0.12 (0.08, 0.16) | 0.16 (0.12, 0.21) | 0.10 (0.07, 0.13) |
| **N** | 29,214 | 26,888 | 29,214 | 26,888 | 24,202 | 19,205 |
